# Disease of the Mouth

**Published:** 1861-09

**Authors:** James E. Garretson

**Affiliations:** Philadelphia


Diseases of the Mouth.—*Osseous Tumors.*—By *James E. Garretson, M. D., of Philadelphia.*—Tumors of the maxillary bones, like tumors of other bones, and like tumors of the soft parts, are benign and malignant, analogous and heterologous.

The maxillary bones, because of this great vascularity and exposure to sources of irritation, are, perhaps, more disposed to enter on pathological conditions, than any other of the ossa coporea.

Malignant tumors of these bones are only local exhibitions of a preëxisting cachexia—diathesis determines diseased action, the result of local injury.

The principal source of irritation to the maxillary bones is found in diseased teeth.

Tumors of these bones, which may, with most assurance, be looked upon as benign, are the Encysted, the Common Inflammatory, and the Exostosis.

Those, the character of which may be esteemed doubtful, if not always malignant, are: the Osteosarcoma (fibro-plastic), Osteo-cephaloma, Osteo-carcinoma, Osteo-melanosis, and Osteo-cancer.

Distinctive diagnostic signs, marking differences between benign and malignant growths, are only appreciable, if indeed appreciable at all, to the very experienced.

Certain general features, however, associate with the two lesions, which may be studied with much advantage.

Benign tumors are mostly referable to a cause, and are apt to be in proportion to that cause.

They are circumscribed by healthy structure. Lymph in a greater or lesser state of organization infiltrates the neighboring tissue, marking the line of demarkation.

They do not seem to have other than the most innocent relation with adjoining organs, so far as disturbance to function is concerned.

The removal of the cause is apt to remove the effect.

In structure, benign tumors are homologous.

They represent the structure of the body to which they are allied. They do not entirely defy constitutional treatment, but are more or less amenable to it.

They are scarcely any other than a single formative capacity.

Malignant tumors are, on the contrary, not at all in proportion to a cause which may be considered the provocative.

They are not healthily circumscribed, but their elementary structure is most apt to be found "infiltrated, inserted, or diffused, in the interspaces and cavities of the tissues in which they lie."

They are associated with a cachexia, which is not unfrequently more marked than the local disorder. Thus, the vis vitæ is not unfrequently so prostrated, that the patient dies outright from the systemic depression. A patient afflicted with cancer has a color, which has not inaptly been likened to a cold buckwheat cake. A cancerous tumor will be found to have affected, markedly, all glands, the vessels running into which have association with it.

The removal of any supposed cause, or the removal of a cancerous tumor itself, will not effect a cure.

In structure, these growths are heterologous; for even if it be contended that, microscopically, their minute histology implies only arrestation of normal development; yet, I think, comparison can not be carried to the varying character of the primary granules, then, (if my pathology is not too old,) are not common to any growth, except the malignant.

True cancer seems not amenable to any constitutional treatment; there are the best of reasons for judging, that all reports of cases cured have origin in mistaken diagnosis.

Malignant tumors have—through the cachexia—a varying formative capacity, they multiply and propagate themselves; if one is removed, a second comes to take its place; either appearing in the site of removal, or in some other locality.

*Benign Osteoids—Cystic Tumor of Paget.*—From Paget, through most surgical writers, mention is made of a slow growing tumor of the jaw, a distinctive pathognomic feature of which is a parchment like crackle given on pressure. These tumors are described as cystic, and are the result of a pouching out, and consequent attenuation of the outer lamina of the bone.

I suppose I have treated, altogether, six or eight of these cases; but I have to offer the singular experience of not having met one of these cysts, which I could make crackle under pressure, or any other manipulation. Depending on this so insisted upon sign, I will recall the trouble and annoyance, and I may add the mortification my first case gave me.

These tumors, as I know them, are slow of growth, being, perhaps, a year in gaining the size of a common hickory nut.* They are, to the touch, as unyielding as the exostoses. The gum covering them is as healthy and natural looking as that in any part of the mouth. Sections of them not unfrequently exhibit septi of bone, which have supported the vault of the tumor, and which, of course, when existing, would prevent any yielding.

*Case.*—Mrs. C——, aged about twenty-one, applied to me, some two years back, for treatment of a tumor occupying the canine fossa of the left superior maxillary bone. The growth had been eighteen months in progress; was about the size of a half walnut, perfectly solid to the touch, painless, and entirely healthy looking; the greatest disquietude of the patient being mental, her mother having died from scirrhus of the rectum.

Associated by marriage, and otherwise, with the family of this lady, are some ten or twelve physicians; each of whom had, in turn, examined the tumor without coming to any satisfactory conclusion concerning it. I am not able now to recall, exactly, how this patient happened to come under my care, but, at any rate, having previously met with two or three precisely similar cases, I felt justified in pronouncing the disease a common cystic tumor; the solidity of which was, not unlikely, attributable to the existence of the septi alluded to.

A choice of operations was suggested to this lady: either, that the cyst should be cut from the body of the bone, or that a crucial incision should be made in it, and the cavity being stuffed with medicated lint, thus obliterated.

Two or three weeks being spent in consultation with friends, the patient was finally submitted to my care, the latter operation being the one decided on.

---

* The reader will, perhaps, recall a similar cystic tumor, of rapid growth, which I described in connection with alveolar abscess. A bony tumor, which sometimes gains the size of a hickory nut in a very few days. (This tumor I believe I am the first to describe.)

The crucial incisions were made; several delicate septi of bone, which the cuts discovered, were broken up, the cyst was injected for the first three days with weak stimulating liquors. No inflammation developing, tufts of cotton were saturated with tinct. of iodine, and the cyst stuffed. In one week the site of the cyst was occupied by healthy granulations; in three weeks the patient was entirely cured, and left the city for her home in an adjoining State.

*Case.*—About nine months back, a Germon woman applied to me with a cystic tumor, similar to the above; it was certainly as unyielding as solid bone. This tumor I treated by making a crucial incision through the soft parts alone, the flaps were dissected off, and the cyst being exposed, was cut away with a chisel-shaped instrument. The flaps fell naturally into the cavity, and were left, even without a stitch, to take care of themselves. The cure was complete in about a week.

The point I would impress, concerning the diagnosis of these tumors is, that the pathognomic crackling, so invariably set down in the books, is not always present; for, as I have remarked, having treated some half-dozen cases, I have not yet felt, or heard it, consequently I have the right, I think, to affirm that it is not a reliable sign.

There is, however, a tumor of the soft parts of the jaw—cystic, but not osseous—which is not to be confounded with the class just described. Both tumors look, occasionally, precisely alike, but the latter yields under pressure, as any tumor of a semi soft part would yield. M. Paget alludes to such a disease in his lectures on Surgical Pathology, pages 342–3. "A woman," he says, "thirty-eight years old, was under my care in 1849, in whom, at first sight, I could not but suppose something was distending the antrum, so closely was deformity of the face due to such disease imitated. But the swelling was soft and elastic, and projected the thin mucous membrane of the gum of the upper jaw, like a half empty sac. I cut into this sac, and let out nearly an ounce of turbid, brownish liquid, sparkling with crystals of cholestearine. The posterior wall of the cyst rested in a deep excavation on the surface of the alveolar border of the upper jaw; an adaptation of shape attained, I suppose, as the result of the long continued pressure of the cyst, which had existed six years."

M. Paget also makes mention of a young man under his care with a similar tumor, which, he says, was the result of an injury to the gum or alveolar border six months previously. In neither of these cases, says he, could I find any disease of the maxillary bone.* Their origin, so far as my experience goes, is in a diseased tooth fang. I do not know what so close an observer as M. Paget means, when he says he could find no disease of the bone. There ought to be at best one little shot-like hole somewhere about the surrounding osseous wall; at any rate, such is the history, as I have met with them.

A succeeding case, which M. Paget mentions, seems to prove that tumors are the same as I refer to. "A lady," he says, "had a small cyst of this kind which had existed twenty-seven years, filling and discharging almost daily. It had its origin in a blow, by which the two median incisors were loosened." This history is that of this particular history of chronic alveolar abscess in every particular.

Last winter, a physician, from Kentucky, applied to me for treatment of a tumor of the lower jaw, which had existed for over two years; it had the feel of a fibroid body. This gentleman, with the imaginative qualities common to the practitioner, when he himself becomes the patient, had succeeded in satisfying himself of the cancerous character of the tumor. An incision through the growth demonstrated it to be a cold alveolar abscess—in a single week he was cured, for the treatment required was, simply keeping patulous the incision, and using a few stimulating injections.

In the *Western Medical Journal*, Vol. X., page 185 to 228, I find a learned article from the pen of Prof. Gross, on "Excision of the Maxillary Bone," in which he makes the following allusion to the osteoid cysts.

"The sero-cystic tumor of the upper jaw is uncommon, and, unfortunately, devoid of malignancy. Its usual site is the alveolar process, where it has been known to attain the volume of a hen's egg, and even of a large orange. It is composed of a thin, semi-transparent, or slightly opaque bag, occupied by a colorless, or sanguineous fluid, or by a glazy, mucilaginous substance. Sometimes, though rarely, there are two such cysts, either closely connected together, or separated by a kind of osseous septum. The bone around the tamor is expanded into a thin, elastic crackling, parchment-

---

* These cysts I have several times met. They are, I think I may affirm cold abscesses.

like shell, and is easily penetrated by a sharp instrument, the puncture giving vent to the characteristic contents of the cyst. This, Professor G. thinks, is the best diagnostic sign of the morbid growth. The general health, he remarks, "remains unaffected in the disease, which is always tardy in its progress, and manifests no disposition to extend among the adjacent structures. Where any doubt exists as to the real nature of the case, he advises a resort to the exploring needle, which, he says, will usually at once dispel it.

Prof. G. thinks it is not often this tumor calls for removal of the affected bone; he gives it as his experience, that in general it will suffice to puncture it occasionally with a small trocar, to evacuate its contents, the escape of which, he says, he has found to be often followed by the rapid contraction and final obliteration of the sac. Where there is a strong tendency to reaccumulation, he recommends that a large opening should be made, and tincture of iodine thrown in, to promote the object in view.

The cystic tumor of Paget is, I presume, the same disease of the jaw to which the name spina ventosa was originally applied—spina to express pain resembling the pricking of thorns, and ventosa, to indicate that the tumor was filled with wind. The term is not yet obsolete, but like epulia, is employed so freely that no one now knows what it means.

*Recapitulatory.*—The cystic tumor of Paget is an osseous cyst of the jaw, which forms just beneath the outer plate of the alveolar, and has its cyst, as a result of the expansion and attenuation of the external lamina.

It is seen as a bulb on the alveolar face, is commonly met with about the size of a half hickory nut, the gum which covers it is perfectly natural in appearance. It may yield to the touch, or it may not.

The most satisfactory, and withal perfectly safe means of diagnosis, is to pass a keen scalpel or lancet into the growth—if it is the tumor we describe, the knife will give to the touch the sense of having passed through a bony wall into a cyst.

Such tumors are treated in two ways: Either by dissecting off from the wall the gum, and chiseling away the cyst; or by opening into the cavity by free incisions, and stuffing with lint—this latter is much the easier operation, indeed, it demands no special operative skill, only requiring that the extent and character of the inflammation, which it is the object to excite, should be watched.

*Exostosis and Inflammatory Tumors.*—These two classes of growths have such parallelisms that their description runs naturally into one another.

The term exostosis, as the reader will recall, is made out of the expressive roots *ex*, out of, and *osteon*, a bone, "an osseous tumor which forms at the surface of bones, or in their cavities." The first is called exostosis—the latter enostosis. The following varieties have been enumerated: Eburnee exostosis; ivory exostosis—that which is ivory-like. Lamina exostosis—that which is made up of distinct fibres, or layers. Spongy exostosis—that which is like the spongy tissue of bone.

Inflammatory tumors of bone are those having a marked origin, as the syphilitic, the scorbutic, the tubercular.

An exostosis proper is strictly benign; it is a disease of exclusively local signification. It is recognizable by its extreme slowness of growth; the entire absence of pain—except where it has some peculiar relation—its freedom from surrounding disease. It is of bony hardness, both to the touch and exploring needle. It does not tend to ulceration, and does not, except mechanically, affect the parts even most directly associated without it.

True exostosis has its origin in local irritation—perhaps always.

It is true that reference is made by authors to an ossific diathesis, but as truly remarked by Miller, "A skeleton so susceptible is prone rather to inflammation and its results:—abscess, ulcers, caries, and necrosis."

I recall a case to which my attention was once directed by my warm and lamented friend, the late Dr. Townsend, dentist. One of the most generous and noble men I have ever known—may his memory long remain green—where an exostosis was evidently the result of a gold filling, which several years before had been inserted into the cavity of a tooth, and which slightly protruded from its neck.

Reflected periodontal inflammation is a common cause of maxillary exostosis, while it must be admitted that cases occur where no primary source of offense is discoverable.

About ten years ago I operated on a medical gentleman for an exostosis situated just below the orifice of the infra orbitol foramen. He could assign no cause for its occurcence.

Exostosis of the fangs of teeth—exostosis dentium—is quite a common disease, and which goes farther than any argument with which I am acquainted to prove an irritative origin of the lesion.

The forms of exostosis occurring here, are the osteo-dentinal and ocemental, and is, of course, peculiar to the region.

While I have seen some two or three cases where the crowns of teeth were enlarged, as if from a species of exostosis, a hypertrophy, yet these were so anomalous, that I may describe the growth as associated exclusively with the fangs, and even here it is mostly confined to the apex, growing as a bulb about the end of the root. Occasionally, however, the whole root is involved, the deposit being quite regularly diffused about it, or the mass may be found as an irregular tubercle on the side of the root.

The diagnosis of exostosis of teeth roots is not always easy. The most frequent pathognomic feature, however, is a sense of continued uneasiness about the parts, not generally amounting to pain, but serving as a constant reminder of the existence of the tooth. The tooth itself may, or may not be carious. Pressure, or the stroke of an instrument does not, in ordinary cases, either increase or diminish the soreness. The sense of fullness about the parts is particularly observed where the absorption of the alveolar is not proportionably active with the oxostosis. In these latter cases a different set of features are provoked.

The pressure is sometimes such as to yield the extremest symptoms of tic douloureux, and patients have been treated weeks for such nervous conditions, where the cause was in this direction. Dr. Harris says the diagnosis (in a case which he describes) was gotten by striking the tooth in a certain direction, a sharp pain shot through the jaw, exactly resembling the former attacks.

One of the most remarkable cases of dental exostosis on record is related by Mr. Fox. The subject was a young lady, who, at the time she sought the professional advice and aid of Mr. Fox, had suffered so severely, and so long, that the palpebræ of one eye had been closed for nearly two months, and the secretion of saliva had for some time been so copious that it flowed from her mouth whenever it was opened. She had tried every remedy which had been recommended by the ablest professional advisers, without realizing any permanent benefit, and she was only relieved from her suffering by the extraction of every one of her teeth.

Mr. S., of Baltimore, having suffered some time from extreme pain in the left superior bicuspis, applied to a dentist in 1843 for the purpose of having this tooth removed. In the operation the root was fractured about three sixteenths of an inch from its extremity, and the upper part left in the socket, and in consequence of which, he did not realize the relief he had hoped to derive from the operation. The pain continued, and at the expiration of twelve months the gum over the upper part of the alveolar became very much swollen, puffing out the lip to the size of half a hen's egg. The tumor after a few days was opened, and a large quantity of dark colored, purulent, and very fetid matter was discharged, which, for a short time, afforded him very considerable relief. The tumor, however, soon reappeared, and was removed some four or five times, by opening it, and discharging the matter in about that number of months.

In the fall of 1845 he called on Dr. Harris, the eminent dental author, for the purpose of obtaining his advice. The gum was swollen, and the lip and cheek protruded as above described. The tumor was again opened, and about three tablespoonfuls of black matter, resembling tar, escaped. Upon further examination the outer wall of the antrum, immediately over the alveolus of the bicuspids which had been fractured, was found destroyed, leaving an opening large enough to admit the end of the forefinger. Believing that the end of the root, which had been left in the socket, was the cause of the disturbance, Dr. Harris urged its immediate removol, and to accomplish this, it became necessary to cut away the outer wall of the alveolus. The root of the tooth, on its removal, was found to be enlarged to the size of a large pea. The secretion of purulent matter soon ceased, and in a few weeks the patient was completely restored.

The following case is from Mr. Bell:

Mr. —— had for some months suffered severe and frequent paroxysms of pain on the left side of the face, apparently commencing in the second inferior bicuspis, and darting through the lower jaw to the ear, and upward to the temple.*

---

*Neuralgic pains in the fifth pair are felt over the forehead, in the lachryma gland, in the eye, the nostril, at the ear, on the neck, in the maxillary bones, about the tip of the tongue, etc. This is explainable anatomically. The nerve rises from the pons varoli, centers at a great ganglia by the ear, then divides into three principal branches. The opthalmic, which passes through the foramen lacerum, and is distributed to the forehead, and by subdivision to the neighboring parts. The superior maxillary branch passing from the cavity of the skull through the foramen rotun-

The pain resembled tic douloureux in the nature of its attacks, but was evidently produced by a local rather than a constitutional cause, from the paroxysms occurring without the least periodical regularity, and from their being excited by the application of heat to the teeth of that part. On the most careful examination, says Mr. Bell, I could not discover the least appearance of caries in any of the teeth, and I therefore ordered leeches to be applied to the gum, and aperient medicines, and abstinence from all stimulating food. This plan was productive of only temporary and partial relief, and in about two days the pain was as severe as ever. Finding that a smart blow on the second bicuspid produced a more painful sensation than on any of the teeth, I determined on extracting it, and found the extremity enlarged by a deposition of bone, giving to it a slightly bulbous shape, but not larger than the tip of a small quill. The removal of the tooth was followed by immediate and entire relief.

Mr. Bell adds, that the newly-formed bone was yellow and more transparent than the original structure.

Many interesting specimens of exostosed teeth roots may be seen in the Museum of the Philadelphia College of Dentistry.—*Medical and Surgical Reporter.*

dum, distributed to the parts which names it. The inferior maxillary, emerging through the oval foramen, subdividing into the lingual branch, which goes to the tongue, and into a motor, which supplies the muscles of mastication. The inferior maxillary nerve supplies the lower jaw and teeth. Neuralgic trismus is explainable by the compound nature of the nerve.

